# Curcumin Supplementation and Endometrial Lining: Examining the Role and Pathophysiology of Use During Frozen-Thawed Embryo Transfer

**DOI:** 10.7759/cureus.20415

**Published:** 2021-12-14

**Authors:** Alexis A O'Connell, Tori E Abdalla, Alexandra A Radulovich, Jordan C Best, Ellen G Wood

**Affiliations:** 1 Research, Alabama College of Osteopathic Medicine, Dothan, USA; 2 Reproductive Endocrinology and Infertility, IVFMD, Cooper City, USA; 3 Biomedical Sciences Program, Philadelphia College of Osteopathic Medicine, Philadelphia, USA; 4 Urology, University of Miami, Miami , USA

**Keywords:** apoptosis, fertility, p53, inducible nitric oxide synthase, estrogen, assisted reproductive technology, endometrial preparation, frozen embryo transfer (fet), turmeric, curcumin

## Abstract

Curcumin is a commonly used herbal supplement purported for its antioxidant, anti-inflammatory, and antineoplastic properties. The effects of curcumin supplementation on endometrial lining have been proposed; however, endometrial preparation in the case of frozen-thawed embryo transfer (FET) has not been established. This case series references two scenarios where turmeric was ingested by the patient, and endometrial thickness was subsequently reduced disrupting the FET cycle. Throughout this case series, curcumin's possible interactions with the uterine lining are summarized. Additionally, these cases highlight the importance of physicians’ awareness of taking a full history of any herbal remedies or supplements in addition to prescription or over-the-counter medications taken when undergoing treatment for controlled FET cycles or in-vitro fertilization (IVF). To our knowledge, no studies to date have investigated this relationship.

## Introduction

Turmeric and its major active component, Curcumin, from the *Curcuma longa* rhizome possess great importance in many cultures, not only as a spice but also as a medical remedy. As it has grown in popularity in western culture, turmeric has become a commercially available health supplement. Purported for its benefits as an anti-inflammatory, antioxidant, antimicrobial, and antineoplastic agent; this herb has been used for centuries and the mechanisms are highly complex [1]. Tumeric has been considered safe at a dosage of 6 grams a day for four to seven weeks; however, extensive studies regarding reproduction, conception, and fertility have not been investigated [2].

During pregnancy, curcumin has been cited as beneficial for mediating oxidative damage and inflammation in pathologies such as preeclampsia, fetal growth restriction, gestational diabetes, and preterm birth [3]. However, the literature referencing dietary curcumin on fertility has shown that by a similar mechanism with which curcumin plays a role in cancer treatment by arresting the tumor cell cycle, enhancing expression of p53, and inducing apoptosis, it can also induce blastocyst apoptosis in early gestation and reduce the success of embryo development and implantation [4,5]. 

Additionally, curcumin modulates estrogen receptivity in many tissues including the uterus, cervix, and breast, and has been used to treat endometriosis by inhibiting the proliferation of epithelial and stromal cells [6]. High doses of curcumin are prescribed in estrogen-dependent breast cancers to inhibit cell proliferation and block the stimulatory effect of exogenous estrogen on estrogen receptor (ER)-positive breast cancer cells [7]. In cervical cancer treatment, curcumin has shown to be effective by inducing apoptosis of tumor cells by altering tissue vascularity [8]. 

Ultimately, the primary outcome of reproductive medicine is intrauterine gestation. Hormonal therapy is a large component of assisted reproductive technology (ART), which acts by simulating the natural cycle to control the timing of physiologic changes. One of several infertility treatments utilized is the frozen-thawed embryo transfer cycle (FET). In this method, a previously frozen embryo is thawed and placed within a uterus to establish pregnancy. Prior to transferring the embryo, an individualized dosage of exogenous estradiol and progesterone hormones are given to establish a thick uterine lining [9]. Because of curcumin’s effects on estrogen receptors, it is possible any supplementation with this herb could interfere with the artificially cultivated environment that FET aims to establish. 

To our knowledge, no studies to date have investigated the impact of curcumin on endometrial preparation for FET. This case series adds to the literature by referencing two cases in which turmeric supplementation was used and endometrial thickness was significantly reduced from optimal endometrial preparation in response to estradiol stimulation.

## Case presentation

Case 1

A 47-year-old Gravida 2 Para 0 female with a past medical history of endometriosis and adenomyosis, underwent preparation for a FET cycle. Prior to arrival at our center, between ages 40 and 42, she failed three prior treatments with in-vitro fertilization (IVF) with her own oocytes. She then elected to proceed to utilize an egg donor and resulted in four total embryos. The first to be implanted was a fresh transfer, and the others were biopsied, frozen, and euploid. Both the initial fresh transfer and a second FET were unsuccessful. 

She had the two remaining euploid embryos transferred to our center. Under our care, the patient underwent a complete evaluation for recurrent implantation failure and pregnancy loss. She had two hysteroscopies during her treatment both revealing a normal cavity with negative pathology and a negative endometrial biopsy. Under our care, she underwent a standard pretreatment protocol of gonadotropin-releasing hormone (GnRH) to inhibit ovulation and transdermal estradiol patches and oral estradiol to prepare the endometrium. Her endometrial thickness was 10 mm in both cycles and estradiol levels were > 200 pg/ml when progesterone support began. The FET resulted in a spontaneous loss at seven weeks where an ultrasound revealed an empty sac, and the second FET cycle was also unsuccessful. She subsequently elected to try again with donor oocytes and created one more euploid embryo.

For her third FET, her endometrium was prepared similarly. However, during this FET preparation, the patient reported feeling particularly ”crampy” secondary to her adenomyosis. She was evaluated during her first monitoring after 12 days of transdermal estrogen patches and oral estradiol. At that time, she had an endometrial lining of 9 mm and estradiol of > 200 pg/ml. Three days after this ultrasound she reported “heavy” vaginal bleeding. She returned to the office for a follow-up ultrasound, revealing a 4 mm endometrial lining and moderate vaginal bleeding. The cycle was canceled due to insufficient lining. 

The patient returned to the office to discuss her next steps. Upon discussion, she admitted that after her first ultrasound she began using “large” scoops of turmeric powder, estimated to be approximately 3000 to 4500 mg, in her morning smoothies per anecdotal evidence to assist with symptoms of severe cramping. She had never done this before in any other FET preparation cycle. She was re-prepared for a FET cycle, excluding turmeric powder without consequence, and underwent a fourth preparation for the existing embryo; however, she suffered another spontaneous loss.

Case 2

**Figure 1:**
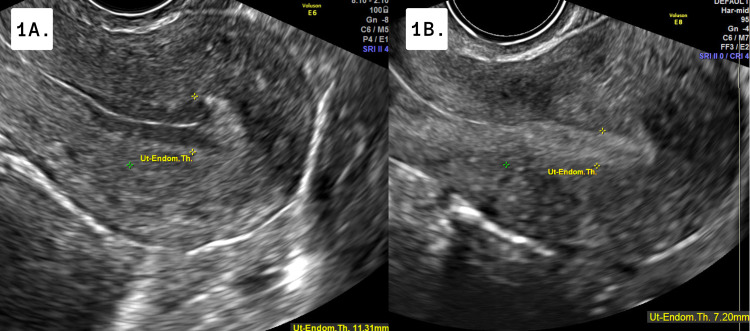
Transvaginal Ultrasound of Endometrial Lining 1A: Image of the adequately prepared endometrial lining of 11.31 mm after exogenous estradiol preparation at the initial sonogram appointment; 1B: Image of endometrial lining of 7.20 mm 7 days status post initial sonogram

A 29-year-old Gravida 2 Para 2 female was undergoing a FET cycle as a gestational carrier to receive a euploid embryo. She was undergoing a standard GnRH pretreatment protocol then using transdermal estradiol patches and oral estradiol. She underwent monitoring after 12 days of estrogen revealing an 11.31 mm grade 2 endometrial lining (Figure 1A) and an estradiol level of 250 pg/mL. She continued the same protocol and returned for her second monitoring. Her endometrial lining changed significantly, shrinking to 7.20 mm with grade 1 features (Figure 1B). The cycle was subsequently canceled. Upon questioning, the patient admitted to drinking “specialty loose tea from a local wellness shop” containing about 1200 mg of turmeric, two to three times a day, starting after her first ultrasound. She was then re-prepared for the next cycle with the same treatment protocol and advised not to ingest any herbs or supplements. She achieved a successful pregnancy for the intended parents in the next FET cycle. 

## Discussion

During ART, embryo transfer relies on adequate endometrial preparation. Standard FET protocols can utilize the natural cycle or hormone replacement therapy (HRT) with GnRH suppression. In HRT, the administration of exogenous estrogen is given during the proliferation phase of the cycle, and progesterone is administered during the secretory phase. In contrast, a natural cycle uses the patient’s endogenous hormones to prepare the endometrium and suppress follicular maturation. After approximately two weeks of priming, transvaginal ultrasonography is then conducted to measure the endometrial thickness and confirm the absence of mature follicles [10]. 

Development of the ideally receptive trilaminar endometrial lining >8 mm on imaging is indicative of an optimally primed lining for implantation due to its prominent outer and central hyperechogenic line and inner hypoechogenic black region [11].

In FET, each millimeter of endometrial thickness ≤7 mm significantly decreases the clinical pregnancy and live birth rates [12]. Practitioners historically have a low threshold for embryo transfer cancellation due to suboptimal endometrial thickness. Once an adequate lining is observed, progesterone supplementation can be started, and FET is scheduled as planned [13].

In the two cases presented, desensitization of the hypothalamic GnRH receptors ensured complete hormonal control of the cycle in preparation for a FET. Estrogen was administered both transdermally and orally. The appropriate endometrial response was noted in both patients as evidenced by an endometrial thickness of 9 mm and 11 mm respectively. However, between the screening ultrasound and follow-up, a notable negative change in the endometrium was observed over a few days reducing the lining in both patients to 4 mm and 7 mm respectively. It is notable that the patient in case 1 was able to proliferate an adequate endometrium in her previous seven transfer cycles, with no evidence of a sudden reduction in the lining. In both cases, the patients denied any deviation to the treatment protocol other than the recent addition of turmeric supplementation in the week between the two scans. Thus, we propose there is a relationship between turmeric supplementation and spontaneous endometrial shedding. 

The proposed mechanism of action of curcumin’s influence on the endometrial lining relates to its many roles in complex signaling pathways. Curcumin inhibits estrogen receptors and reduces tissue vascularity, potentially negatively impacting the endometrial preparation for FET [14]. One significant mediator is curcumin’s inhibition of inducible nitric oxide synthase (iNOS). iNOS is present in large quantities in the secretory endometrium glandular epithelial cells. Normally, it has been shown to increase the effect of estrogen and progesterone stimulation during the menstrual cycle and along with decidualized endometrium during pregnancy controlling tissue vascularity [15,16]. Inhibition of iNOS enzyme in the uterine lining could acutely alter tissue perfusion impairing vasodilation and angiogenesis to the endometrium [17, 18].

Additionally, curcumin’s potential to reduce the production of estradiol and its effects on the epithelial and stromal cells have been extensively studied in the setting of endometriosis [6]. The downstream effects of estrogen are blunted by the impaired receptor activity although serum estradiol may remain normal due to artificial and residual estrogen. By a similar mechanism, curcumin’s therapeutic effect on both endometrial and breast cancer functions by targeting key transcription factors including nuclear factor kappa light chain enhancer of activated B cells (NFkB), phosphatidylinositol-3-kinase (PI3K)/Akt/mammalian target of rapamycin (mTOR), mitogen-activated protein kinase (MAPK), Janus kinase/signal transducers and activators of transcription (JAK/STAT), and microRNA to alter the cell cycle, causing apoptosis and impaired angiogenesis [19].

Further, curcumin enhances the expression of p53, a cell cycle mediator, implicated in the pathophysiology of many malignant tumors including cervical cancer, high-grade ovarian carcinoma, endometrial cancer, and even colorectal cancer particularly in a time and dose-dependent manner [20,21].

Because increased p53 activity can speed up tissue apoptosis, it is probable that during the acute proliferation of endometrial lining in the case of intentional hormone stimulation, cells in the endometrium respond like tumor cells. Because of the low oral bioavailability and solubility, a dose of turmeric greater than 10,000-12,000 mg is necessary for a measurable therapeutic effect, but doses greater than 1500 mg during FET may affect endometrial preparation [20].

We suggest any concentration of curcumin during FET may affect endometrial preparation. The impact on the endometrial receptivity of implanted embryos remains a concern. In the cases discussed herein, the physician discontinued the treatment at the observation of reduced endometrial lining with high clinical suspicion of a potentially failed transfer. This cancellation of the treatment regimen due to insufficient endometrial preparation resulted in a financial, emotional, and physical burden to the patients at hand. In both cases, a donor egg embryo was used, and thus the window of opportunity for ART was limited.

This case report possesses multiple limitations that should be addressed. The self-reported account of the patients' dietary habits is limited by the subjective nature of the data itself. The presumed connection between turmeric and the potential adverse effects seen during endometrial preparation is key in this investigation. In addition, many confounding factors were not able to be controlled, such as the impurity or contamination of the turmeric supplement in both cases, other herbals that might have been included in both cases, that the patients have not disclosed.

Despite all limitations, these two scenarios highlight the importance of health care practitioners conducting a holistic review of the patient’s medical history to not only include medications, but also herbals, supplements, and vitamins. These interventions are not benign and may be directly involved in pathologies observed. Several other supplemental micronutrients have been found to impact fertility treatments including folic acid, vitamin D, long-chain omega 3 fatty acids, and Coenzyme Q10 (CoQ-10) with an increase in overall fertility and decreased time to conceive [22]. 

The mechanisms behind curcumin’s impact on the fertility cycle are complex and not fully understood. Existing studies have not clearly established a mechanism responsible for the changes observed in our two patients. More research is necessary to determine the safety and efficacy of modern turmeric supplementation in FET preparation and early pregnancy. As these herbal supplements become more popular, it is apparent that physicians may need more comprehensive education on the mechanisms of these active substances to provide competent medical counseling addressing interactions and harms for patients interested in adding these to their daily regimen. 

## Conclusions

The case report offers insight into the potentially detrimental effects of turmeric supplementation on endometrial preparation during fertility treatment. These two case presentations serve as an example of the relationship between turmeric and the endometrial lining. Unfortunately, our data is limited by the subjective nature of our patient's disclosure of medication compliance and herbal supplement use.

As a retrospective observational study, we were not able to control for confounding variables and or examine the quality of the supplement they consumed. Thus, the need for experimental studies to examine if there is a causal role of curcumin on the endometrial lining and its role in endometrial receptivity in human subjects. Most of the existing literature investigates curcumin’s role in modulating estrogen receptivity regarding endometriosis and cancer and uses murine subjects. There is a dearth of literature surrounding curcumin’s role during fertility treatments and early gestation.

Therefore, we recommend that the effects of turmeric supplementation on fertility and pregnancy be further studied. In addition, these two cases illustrate the importance of gaining a holistic and comprehensive medical history of the patient, including herbal supplements and vitamins, to achieve optimal outcomes, not only in infertility treatments but in all aspects of medicine. 
